# Differences in Cellular Clearing Mechanisms of Aggregates of Two Subtypes of HLA-B27

**DOI:** 10.3389/fimmu.2021.795053

**Published:** 2022-01-10

**Authors:** Amit Kumar Thakur, Manni Luthra-Guptasarma

**Affiliations:** Department of Immunopathology, Postgraduate Institute of Medical Education and Research (PGIMER), Chandigarh, India

**Keywords:** HLA-B27 alleles, high molecular weight (HMW), aggregates, clearance, proteomics

## Abstract

Ankylosing spondylitis (AS) belongs to a group of diseases, called spondyloarthropathies (SpA), that are strongly associated with the genetic marker HLA-B27. AS is characterized by inflammation of joints and primarily affects the spine. Over 160 subtypes of HLA-B27 are known, owing to high polymorphism. Some are strongly associated with disease (e.g., B*2704), whereas others are not (e.g., B*2709). Misfolding of HLA-B27 molecules [as dimers, or as high-molecular-weight (HMW) oligomers] is one of several hypotheses proposed to explain the link between HLA-B27 and AS. Our group has previously established the existence of HMW species of HLA-B27 in AS patients. Still, very little is known about the mechanisms underlying differences in pathogenic outcomes of different HLA-B27 subtypes. We conducted a proteomics-based evaluation of the differential disease association of HLA B*2704 and B*2709, using stable transfectants of genes encoding the two proteins. A clear difference was observed in protein clearance mechanisms: whereas unfolded protein response (UPR), autophagy, and aggresomes were involved in the degradation of B*2704, the endosome–lysosome machinery was primarily involved in B*2709 degradation. These differences offer insights into the differential disease association of B*2704 and B*2709.

## Introduction

Studies over the last two decades have helped to understand the link between the strong association of HLA-B27 and ankylosing spondylitis (AS). It has been established that the heavy chain of HLA-B27 has a tendency to misfold through the formation of either disulfide-linked dimers or oligomers/high-molecular-weight (HMW) species (1–3). Such misfolding events can occur in the endoplasmic reticulum (ER) prior to the assembly of the HLA trimer [consisting of the HLA heavy chain, the β2 microglobulin (β2m) chain, and bound nonameric peptide] to generate ER stress, together with activation of the unfolded protein response (UPR) and the subsequent activation of macrophages to produce cytokines causing inflammation (4). Misfolded forms of HLA-B27 have been observed on cell surfaces, in the form of β2m-free homodimers. These are believed to cause pathology through binding with killer immunoglobulin-like receptors (KIRs) and leucocyte immunoglobulin-like receptors (LILRs), or through deposition within synovial tissues, resulting in activation and regulation of the immune system (5).

Previously, we have proposed that β2m-free heavy chains of HLA-B27 can undergo a facile conformational change to allow a region of its own chain to bind to either the peptide-binding cleft of the same polypeptide chain (self-display) or the cleft of another polypeptide chain (cross-display). The latter was proposed to lead to the formation of large, soluble, HMW, degradation-resistant, long-surviving aggregates of the HLA-B27 heavy chain (6). We and others have also shown the existence of these HMW aggregates or oligomers of HLA chains in cells transfected with HLA-B27, as well as in AS patients (7, 8).

Although the above work provided clues explaining the link between the misfolding of HLA-B27 and AS, there are scanty data to explain the differential association of HLA-B27 subtypes with AS. Interestingly, despite the level of oligomerization in the disease-associated and non-disease-associated HLA-B27 subtypes being similar (3), the former differs from the latter by an increased tendency to accumulate in intracellular ER-derived vesicles, leading to ER-associated degradation (ERAD) of the heavy chains and the UPR, thereby causing upregulation of the proinflammatory cytokines (8).

Differential stabilities and half-lives of HMW species associated with the disease-associated HLA B*2704 and non-disease-associated HLA B*2709 subtypes have been noted by us (unpublished data). These HMW species might be anticipated to pose a problem for cellular machinery, in terms of mechanisms (especially quality control (QC) mechanisms) for their disposal. Therefore, we considered it necessary to evaluate whether cells transfected with disease-associated and non-disease-associated subtypes of HLA-B27 recruit different cellular machineries (QC processes) for their degradation and turnover.

Here, we provide evidence-based on proteomics and other molecular and cellular correlates to indicate that the disease-associated subtype, HLA B*2704, is mainly disposed off through activation of the UPR and activation of autophagy and the involvement of aggresomes. In contrast, the non-disease-associated subtype B*2709 is mainly disposed off through the endosome–lysosome machinery.

## Materials and Methods

### Generation of Stable Transfectants of Full-Length Subtypes

One lakh cells (H1299) were cultured in complete growth media (Dulbecco’s modified Eagle medium (DMEM) high glucose with 10% fetal bovine serum (FBS)), and following confluency of ~70%, cells were plated in serum-free growth media without antibiotics overnight. Cells were then individually transfected with full-length cDNA constructs of each of the two subtypes (B*2704 and B*2709, cloned in pEGFP plasmid, with green fluorescent protein (GFP) in fusion in the C-terminus of the HLA gene), using Lipofectamine™2000 (Invitrogen; Cat No. 11668019). After 6 h of transfection, complete media were added, i.e., DMEM (high glucose) supplemented with 10% FBS, and cells were grown further for 24 h. Stable transfectants were generated by plating cells with serial dilution onto a 96-well plate in DMEM (high glucose) + 10% FBS containing different concentrations of geneticin (200–800 μg/ml). Single-cell clones were selected and then were further grown in complete media.

### Sample Preparation for Liquid Chromatography–Mass Spectrometry

Cells were detached using trypsin (cell culture, Gibco^®^) and centrifuged at 2,000 rpm for 5 min. The supernatant was discarded, and the pellet was washed using 1× TBS (50 mM of Tris-Cl, pH 7.5; 50 mM of NaCl), followed by the addition of protease inhibitors (Sigma; Cat No. P8340). Complete cell lysis was performed using 6MGnHCl (guanidine hydrochloride), followed by sonication and heating at 90°C for 5 min; lysate was centrifuged at 15,000 rpm for 20 min, and the supernatant was used for protein estimation by the bicinchoninic acid (BCA) protein assay kit (Sigma Aldrich, Inc.). Samples were reduced and alkylated with 10 mM of dithiothreitol (DTT; Sigma-Aldrich) and 50 mM of iodoacetamide (IAA; Sigma-Aldrich), respectively, for 1 h in the dark. The pH of diluted samples (10 times) was adjusted to 8–8.5 and trypsinized (Trypsin, Sigma-Aldrich) at 1:20 enzyme to protein ratio for 16 h at 37°C. Before sample clean-up using C18 clean-up columns, pH of each sample was adjusted to around 2 using 10% trifluoroacetic acid (TFA), followed by vacuum drying of samples and reconstitution using 0.1% formic acid. The protein concentration of each sample was determined using NanoDrop (Thermo Fisher Scientific) spectrophotometer, and equal amounts were loaded for liquid chromatography–mass spectrometry (LC-MS) analysis.

### Mass Spectrometric Analysis of Peptide Mixtures

Tryptic peptides measuring 1 µg of each sample were analyzed using Orbitrap Fusion™ Tribrid™ Mass Spectrometer (Thermo Scientific, USA) coupled to EASY-nLC 1200 system equipped with nanoelectrospray ion source. Peptides were resolved on nano-LC reverse phase column (75 μm ID × 25 cm, packed with PepMap 2-μm C18 particles) for 95 min with a gradient of 5%–45% acetonitrile in 0.1% formic acid at a flow rate of 300 nl/min. MS1 survey scans were performed with a resolution of 120,000 and a mass range of 375–1,600 *m*/*z*, on the Orbitrap. Peptides with charge states 2–5 were sampled for MS2. Following higher-energy C-trap dissociation (HCD) activation, tandem MS (MS/MS) data were acquired using the ion trap analyzer in centroid mode. MS was operated in data-dependent acquisition mode using 30% HCD collision energy and automatic gain control (AGC) target of 5.0e5. Lock mass option was enabled for polydimethylcyclosiloxane (PCM) ions (*m*/*z* = 445.120025) for internal recalibration during the run. The MS/MS data have been deposited to the ProteomeXchange Consortium *via* the PRIDE partner repository (9) with the dataset identifier PXD027999.

### Data Processing

The raw files generated were used for protein identification using Proteome Discoverer 2.4 (Thermo Fisher Scientific Inc., Austria) by searching against a standard *Homo sapiens* database from UniProtKB *Homo sapiens* database UP000005640 using Sequest HT search engine with 20289 sequences. Sequest HT search criteria included tryptic cleavage with two missed cleavages, a precursor mass tolerance of 10 ppm, and fragment mass tolerance of 0.6 Da. Minimum peptide length was set at 6, and maximum peptide length was 144. Search criteria also included carbamidomethylation of cysteines as static modification and oxidation of methionine as a dynamic modification. Default settings were used for other parameters. Protein identification and quantitative analysis used more than two unique peptides; false discovery rate (FDR) of less than 1% and p-values <0.05 were required for relative quantification.

### Bioinformatics Analysis

For the purposes of relative quantification analysis, we included all the proteins above 1.5 fold change. Pathway analyses were done using Database for Annotation, Visualization and Integrated Discovery (DAVID) (https://david.ncifcrf.gov/) and Integrated Molecular Pathway Level Analysis (IMPaLA) software. Boxplot and heat map were generated using Proteome discoverer 2.4. A principal component analysis (PCA) plot was generated using R software version 3.4.1. Functional network construction of protein–protein interactions (PPIs) was performed by STRING version 11 network.

### Western Blotting

Stable transfectant cells of B*2704 and B*2709 were seeded in 6-well plates containing complete DMEM with 10% FBS; post 70%–80% confluency, cells were serum starved for 6 h and then treated with 50 μM of chloroquine (CQ), 100 nM of bafilomycin (Baf), or combination (CQ+Baf) for 12 h. Similarly, cells were seeded, serum starved for 6 h, and then treated with proteasomal inhibitor MG132 (15 μM) for 4 h and with the UPR inducer thapsigargin (TG) measuring 1 μM for 24 h. After treatment, cells were washed with 1× phosphate-buffered saline (PBS) and then trypsinized. Cells were pelleted after centrifugation at 2,000 rpm for 5 min, washed with 1× PBS, followed by lysis using radioimmunoprecipitation assay (RIPA) buffer (50 mM of Tris HCl, 1% Triton, 0.5% sodium deoxycholate, 0.1% sodium dodecyl sulfate (SDS), 2 mM of EDTA, 150 mM of NaCl, and a cocktail of protease inhibitors). Following incubation on ice for 30 min, centrifugation was performed at 12,000 rpm for 20 min at 4°C. The supernatants were collected and mixed with Laemmli loading buffer; equal amounts of sample (approx. 40 μg) were loaded onto 10% or 12% SDS–polyacrylamide gel electrophoresis (PAGE) gels for analysis of heavy chains and p62 or LC3, respectively. Gels (10%) were also run for cells treated or untreated with CQ, under non-reducing conditions (with SDS in the running buffer), to evaluate the presence of oligomeric species by Western blotting using the HC10 antibody. Blocking was done in 5% skim milk for 2 h at room temperature, before developing the blots using the following primary antibodies, incubated overnight at 4°C: heavy chain-specific HC10 antibody for detection of heavy chains and anti-LC3 (Novus Biologicals; Cat No. NB600-1384) and anti-p62 (Novus Biologicals; Cat No. NBP1-48320) antibodies for detection of autophagy markers. Mouse anti-horseradish peroxidase (anti-HRP) antibody (Invitrogen; Cat No-A0168-1ML) was used as secondary antibody for HC10 staining, and rabbit anti-HRP antibody (Invitrogen; A0545-1ML) was used for probing LC3 as well as p62. Blots were developed using Clarity Western ECL substrate (Bio-Rad; Cat No. 170-5061). Quantitation of signal intensities was performed using the ImageJ software and normalized against the house-keeping β-actin (Sigma; Cat No. A1978).

### Confocal Microscopy

H1299 cells and stable transfectant cells of B*2704 and B*2709 were seeded on 24-well plates over coverslips. After approximately 50% confluency, cells were left untreated or treated with CQ (50 μM) or Baf (100 nM). After treatment, cells were fixed with 4% paraformaldehyde, permeabilized with 0.3% Triton-X 100, and blocked with 1% bovine serum albumin (BSA) for 30 min. Cells were incubated overnight at 4°C with primary antibodies against LC3, p62, and vimentin (Sigma; Cat No. V6389); anti-rabbit Alexa Fluor 568 (Invitrogen; Cat No A11011) was used as secondary antibody for visualizing LC3 and p62; and anti-mouse Alexa Fluor 594 (Invitrogen; Cat No. A11032) was used for imaging vimentin. Nuclear staining was done with DAPI. Images were acquired using an Olympus microscope. Images were analyzed using Fiji software. Colocalization was determined using ImageJ software by taking Pearson’s coefficient value.

### Real-Time PCR

H1299 and stable transfectant cells of B*2704 and B*2709 were seeded in 24-well plates, in the presence and absence of CQ. RNA was isolated using RNeasy mini kit (Qiagen), and cDNA was prepared using verso cDNA synthesis kit (Qiagen). One microliter of cDNA was added to each PCR master mix (Promega) (20 µl), containing 0.25 µM of each primer and 10 µl of 2× iTaq SYBR Green supermix (Bio-Rad Laboratories, Hercules, CA). The following protocol was used: 35 cycles of denaturation step at 95°C for 30 s, annealing at 60°C for 1 min, followed by extension at 72°C for 30 s, with a final standard dissociation protocol to obtain the melting profiles. The gene expression of various genes was evaluated using quantitative real-time PCR (Roche 480). Expression levels in non-transfected cells were used as the baseline. Relative quantification of the targets in each sample was carried out using the signal of GAPDH as a control. Relative gene expression levels were calculated according to the 2^−ΔΔCt^ method.

## Results

### Identification of the Differentially Expressed Proteins in Cells Stably Transfected With Disease-Associated (B*2704) and Non-Disease-Associated (B*2709) Subtypes of HLA-B27

The NCI-H1299 (human non-small cell lung carcinoma cell line) cells (HLAA*2402, 3201; HLAB*4002, 4002) were used for the transfection of the two subtypes of HLA-B27, with the GFP fused to the C-terminus of each subtype. Previously, other cancer cell lines (such as HeLa cells) transfected with HLA-B27 alleles (carrying fluorescent molecules at their C-terminal) have been similarly used as experimental cell models to study their intracellular trafficking (3). Label-free quantitation of stable transfectants of H1299 cells carrying disease associated, HLA B*2704, and non-disease associated, HLA B*2709, was carried out. This was done to examine protein QC mechanisms operating in respect of the two subtypes towards clearance of protein aggregates and maintenance of cellular homeostasis. A boxplot of protein abundances in 12 samples, composed of 6 samples each for the transfection-based expression of B*2704 and B*2709, is depicted in **Figure S1A**. The PCA data are presented in **Figure S1B**, illustrating the sample distribution of six B*2704 (red) and six B*2709 (green) samples, showing a clear separation of the two types of samples, based on 1,672 proteins (**Table S1**).

**Figure 1:**
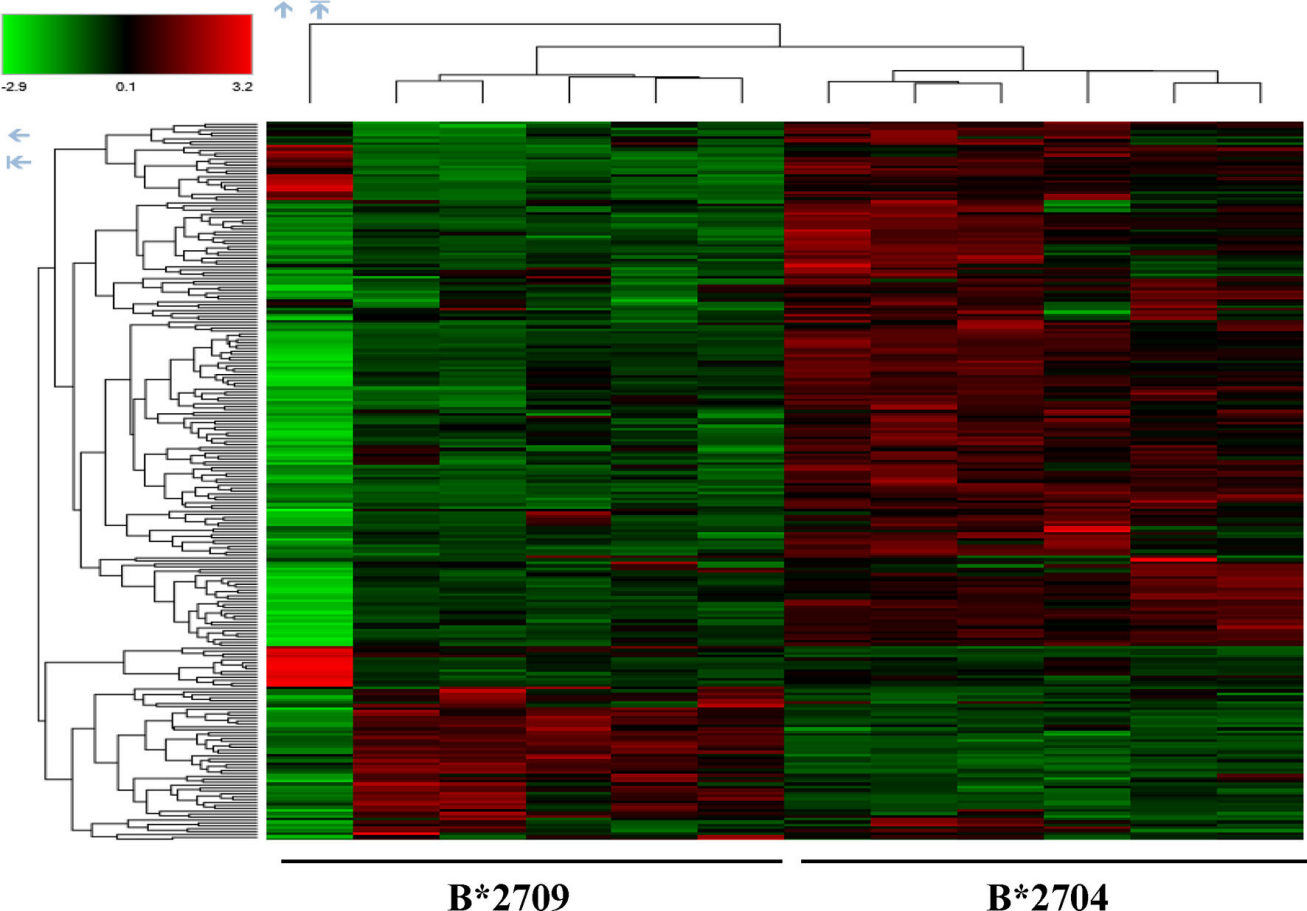
Hierarchical clustering and heat map based on label-free proteome quantification, representing color-coded expression levels of differentially expressed proteins in cells transfected with the two subtypes of HLA-B27. Red color represents the upregulated proteins, while green shows the downregulated proteins in the two cell types, with 6 biological replicates in each case.

**Figure 2:**
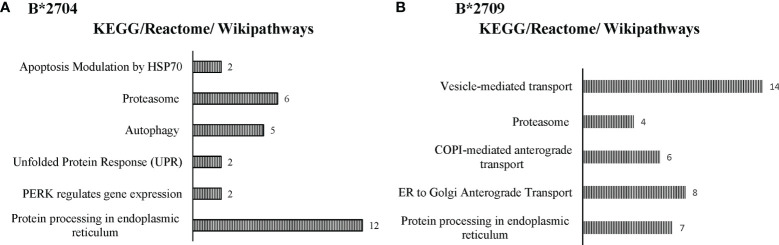
Enriched biological pathways, along with respective protein counts (shown as numbers at the bar-edges) for proteins seen to be differentially upregulated in cells expressing B*2704 **(A)** and B*2709 **(B)**.

Further, hierarchical clustering analysis (**Figure 1**), with the relative abundance of each protein being color-coded and based on the z-score of the protein’s normalized peak area), shows that of the 1,672 proteins detected and quantified in the cell extracts of the two subtypes, 261 proteins were upregulated in B*2704 samples compared with B*2709 samples, and 174 proteins were upregulated in the B*2709 samples compared with B*2704 samples (**Tables S2, S3**, respectively). The fold change was calculated from normalized abundances of proteins in both subtypes, and the UniProt accession numbers of proteins that are differentially upregulated (>1.5 fold) in the two subtypes (**Tables S2, S3**) were independently uploaded on DAVID (version 6.8) and IMPaLA (version 12) software, to examine enriched pathways contributed by the differentially expressed proteins in each subtype. In addition to unique pathways in the two subtypes of HLA-B27, the pathway analysis (**Figure 2** and **Tables S4, S5**) reveals some pathways related to protein clearance that are common to these subtypes, including protein processing in the ER and the proteasome. The list of proteins involved in these 2 pathways is presented in **Tables 1**, **2** for both B*2704 and B*2709, respectively.

**Table 1:** Statistically significant differentially expressed proteins classified under the KEGG pathway, protein processing in ER.

Protein name	UniProt ID	Mol. mass (kDa)	Unique peptides	Coverage (%)	Fold change
Proteins upregulated in B*2704 relative to B*2709					
B-cell receptor-associated protein 31 (BCAP31)	P51572	28	2	13	2.26
BCL2 associated athanogene 2 (BAG2)	O95816	23.8	6	48	2.03
DnaJ heat shock protein family (Hsp40) member B1 (DNAJB1)	P25685	38	3	22	1.61
DnaJ heat shock protein family (Hsp40) member B11 (DNAJB11)	Q9UBS4	40.5	3	11	1.52
Endoplasmic reticulum oxidoreductase 1 alpha	Q96HE7	54.4	3	112	1.59
Eukaryotic translation initiation factor 2 subunit alpha	P05198	36.1	10	36	1.68
Heat shock protein family A (Hsp 70) member 1A (HSPA1A)	P0DMV9		23		1.59
Heat shock protein family A (Hsp 70) member 5 (HSPA5)	P11021	72.3	30	44	1.67
Heat shock protein family A (HSPHA 110) member 5 (HSPH1)	Q92598	96.8	23	41	1.80
Ubiquilin-1 (UBQLN1)	Q9UMXO	62.5	2	9	1.62
Ubiquilin-2 (UBQLN2)	Q9UHD9	65.7	3	8	1.50
Proteins upregulated in B*2709 relative to B*2704					
Sec 13 homolog, nuclear pore and COPII coat complex component (Sec 13)	P55735	35.5	2	11	2.73
Sec 31 homolog A, COPII coat complex component (Sec 31A)	O94979	132.9	3	4	2.08
Calnexin (CANX)	P27824	67.5	9	24	1.54
Heat shock protein family A (Hsp70) member 4 like (HSPA4L)	O95757	94.5	7	17	1.72
Hypoxia upregulated (Hyou1)	Q9Y4L1	111.3	11	21	1.64
Protein disulfide isomerase family A (PDIA4)	P13667	72.9	13	26	1.78
Ribophorin II (RPN2)	P04844	69.2	8	25	1.63

KEGG, Kyoto Encyclopedia of Genes and Genomes; ER, endoplasmic reticulum.

**Table 2:** Statistically significant, differentially expressed proteins classified under the KEGG pathway, “Proteasome”.

Protein name	UniProt ID	Mol. mass (kDa)	Unique peptides	Coverage (%)	Fold change
Proteins upregulated in B*2704					
Proteasome 26S subunit, ATPase 1 (PSMC1)	P62191	49.2	8	24	1.62
Proteasome 26S subunit, ATPase 3 (PSMC3)	P17980	49.2	8	30	1.55
Proteasome 26S subunit, ATPase 6 (PSMC6)	P62333	44.1	8	31	1.61
Proteasome 26S subunit, non-ATPase 2 (PSMD2)	Q13200	29.5	5	25	1.64
Proteasome activator subunit 1 (PSME1)	Q06323	28.7	3	15	1.55
Proteasome subunit alpha 1 (PSMA1)	P25786	29.5	5	25	1.56
Proteins upregulated in B*2709					
Proteasome 26s subunit, non-ATPase 1 (PSMD1)	Q99460	105.8	5	11	1.63
Proteasome subunit beta 4 (PSMB4)	P28070	29.2	6	44	1.54
Proteasome subunit beta 7 (PSMB7)	Q99436	29.9	3	32	2.08

KEGG, Kyoto Encyclopedia of Genes and Genomes.

Interestingly, in the case of B*2704, in addition to the above pathways, “autophagy” appeared among the top 25 annotation clusters (using the DAVID software) with a group enrichment score greater than 0.6 (p = 0.08). Further, using the IMPaLA software too, autophagy was one of the Kyoto Encyclopedia of Genes and Genomes (KEGG) pathways suggested for B*2704 (p = 0.06). The list of proteins implicated in the process of autophagy as indicated by these two software is presented in **Table 3**. The possibility of involvement of autophagy in B*2704 approached the borderline of significance, as suggested by both software independently. We decided, therefore, to investigate whether autophagy is indeed involved in the clearance of HMW aggregates in this subtype, by carrying out separate confirmatory experiments, described later.

**Table 3:** List of proteins associated with autophagy and retrograde transport, identified to be upregulated in B*2704-transfected cells.

Protein name	Gene symbol	UniProt ID	Mol. mass (kDa)	Unique peptides	Coverage (%)	Fold change	Software
RAB7A, member RAS oncogene family (RAB 7A)	RAB7	P51149	23.5	5	31	1.54	DAVID/IMPaLA
Sequestosome-1	SQSTM1	Q13501	47.7	13/54	54	1.50	DAVID
Synaptosomal-associated protein 29 (SNAP29)	SNAP29	O95721	29	2	14	3.41	DAVID/IMPaLA
Ubiquilin-1 (UBQLN1)	UBQLN1	Q9UMXO	62.5	2	9	1.62	DAVID
Ubiquilin-2 (UBQLN2)	UBQLN2	Q9UHD9	65.7	3	8	1.50	DAVID
Microtubule-associated protein 1B		P46821	270.5	5	5	2.04	DAVID
Early endosome antigen 1	EEA1	Q15075	162.4	4	5	1.63	DAVID
Dynein light chain roadblock-type 1 DYNLRB1	DYNLRB1	Q9NP97	10.9	3	34	1.54	DAVID
Dynamin-binding protein DNMBP	DNMBP	Q6XZF7	177.2	4	5	1.54	DAVID

DAVID, Database for Annotation, Visualization and Integrated Discovery; IMPaLA, Integrated Molecular Pathway Level Analysis.

To examine potential interactions between differentially expressed proteins in each of the two subtypes, the STRING tool was employed. **Figures S2**, **S3** (left panels) show only the physical network, in an effort to minimize the network cluster; the edges indicate that the proteins are part of a physical complex (without signifying functional association). The panel on the right of **Figure S2** (B*2704) shows that some of the proteins upregulated in B*2704 are engaged in PPIs in the processes of UPR and autophagy. The differentially expressed proteins in B*2709, on the other hand, belong to the trans-Golgi network, or the endosomal–lysosomal pathway (**Figure S3**; right panel).

The differentially expressed proteins belonging to the pathway, “protein processing in ER” (obtained from DAVID software, in both subtypes), were also uploaded in the STRING software to determine the PPIs, as well as to deduce the contribution of the individual (upregulated) proteins present in the ER lumen, which facilitates three types of protein processing: a) protein folding with the help of lumenal chaperones and packaging into transport vesicles, to transport them to the Golgi complex; b) binding of BiP to the terminally misfolded proteins, to direct them towards degradation through the proteasome (ERAD); or c) accumulation of misfolded proteins in the ER, causing ER stress and activation of the UPR signalling pathway. **Tables S6, S7** list the biological processes as well as cellular components involved in “protein processing in ER” as determined using the STRING analysis in B*2704 and B*2709, respectively. Notably, the biological processes associated with the differentially expressed proteins in the case of B*2704-transfected cells included the UPR, response to ER stress, cellular response to oxidative stress, and regulation of the intrinsic apoptotic pathway (**Table S6**), whereas in the case of B*2709-transfected cells, the processes included ER to Golgi vesicle-mediated transport including COPII-coated vesicle cargo loading and receptor-mediated endocytosis (**Table S7**), suggestive of the endosomal–lysosomal pathway (10, 11).

When the autophagy-related proteins (derived from DAVID software analysis, in the case of B*2704) were uploaded into the STRING software, it was seen that the processes included macroautophagy and selective autophagy, with the cellular components including the autophagosome, phagophore assembly site, and autophagosome membrane (**Table S8**).

In addition, use of the Gene Ontology software (http://geneontology.org/) led us to another possible pathway, i.e., aggresomal pathway, operating in the case of B*2704, with upregulation of proteins involved in retrograde transport and proteins characteristic of aggresomes, including proteins belonging to the BAG family (such as molecular chaperone regulator 3 or BAG3), sequestosome-1 (SQSTM1) and ubiquilin-1 (listed in **Table 3**) (12–14).

The overall picture that emerged from the above data is that unique pathways operate in respect of the processing of misfolded HLA chains of the two subtypes, in cells expressing them due to transfection. Proteomics-based analyses suggest clearance of aggregates through UPR, autophagy, and aggresomes in the case of B*2704 and the endosomal–lysosomal pathway in the case of B*2709.

### Confirmation of Macroautophagy in B*2704-Transfected Cells

**Figure 3:**
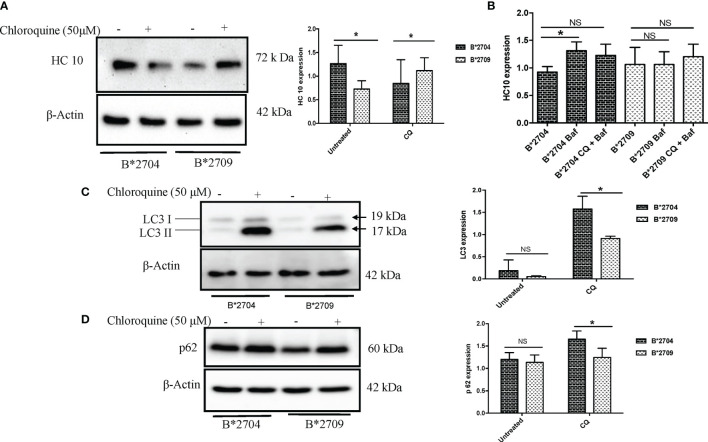
Stable transfectants of pEGFP-HLA-B27 subtypes (B*2704 and B*2709), in the absence (untreated) or presence of CQ, were probed with HC10 antibody **(A)** and also treated with Baf and Baf+CQ **(B)** for detection of heavy chains. Expression levels of autophagy markers LC3 **(C)** and p62 **(D)** were evaluated. Western blotting was done, and actin levels were probed to serve as loading control for all samples to allow normalization of the signals. In each panel, histograms show densitometry data (mean ± SD) from 3 experiments corresponding to the Western blotting, using ImageJ software. Baf, bafilomycin; CQ, chloroquine. NS, non significant (p > 0.05); *p < 0.05.

To confirm the involvement of macroautophagy in cells expressing B*2704 through transfection (as compared with cells expressing B*2709), we used the autophagy inhibitor CQ (50 μM). The presence of aggregates was checked by using the HLA heavy chain-specific mAb, HC10, and Western blotting. The presence of autophagy markers LC3 and p62 was also examined by Western blotting. In the HLA-B27-transfected, CQ-untreated (−CQ) cells, the expression of HC10 was significantly more in B*2704-transfected cells as compared with cells overexpressing B*2709 (**Figure 3A**), suggestive of increased expression of misfolded forms of HLA-B27 in B*2704-transfected cells.

Treatment with CQ caused significantly decreased HC10 binding (p = 0.03) in B*2704-expressing cells, unlike cells transfected with B*2709, which correlates with a corresponding decline and rise in oligomeric species, as seen under non-reducing SDS-PAGE conditions (**Figure S4**). However, surprisingly, significantly increased expression of autophagy markers LC3 and p62 was evident in B*2704-expressing cells, as compared with B*2709-expressing cells (**Figures 3C, D**). It would be expected that inhibition of autophagy (through CQ treatment) would result in a corresponding accumulation of heavy chains in B*2704-transfected cells, but on the contrary, we observed a decrease in HC10 binding. Considering the possibility that the aggregates in B*2704-expressing cells can possibly relieve the stress through other alternative pathways such as UPR, we examined the levels of UPR-related genes such as ATF4, BiP, and CHOP, consequent to CQ treatment; as expected, the levels of all three genes were significantly increased for B*2704-transfected cells upon treatment with CQ for 12 h (**Figure S5**). Therefore, we propose that the UPR pathway is responsible for the clearance of proteins following autophagic inhibition, leading to reduced accumulation of HC10 reactive species.

Further, we wished to use another autophagy inhibitor, Baf, which is a potent inhibitor of the Vacuolar H+ATPase (15), which controls lysosomal pH, as well as a combination of CQ and Baf to observe the convergent effects on the accumulation of heavy chains as well as autophagy markers. Clearly, a 12-h treatment of B*2704-expressing cells with Baf alone led to a significant increase (p = 0.02) in the expression of HC10 reactive species, and a combination of the two inhibitors also showed an increase, although the increase was not significant (p = 0.07) (**Figure 3B**).

**Figure 4:**
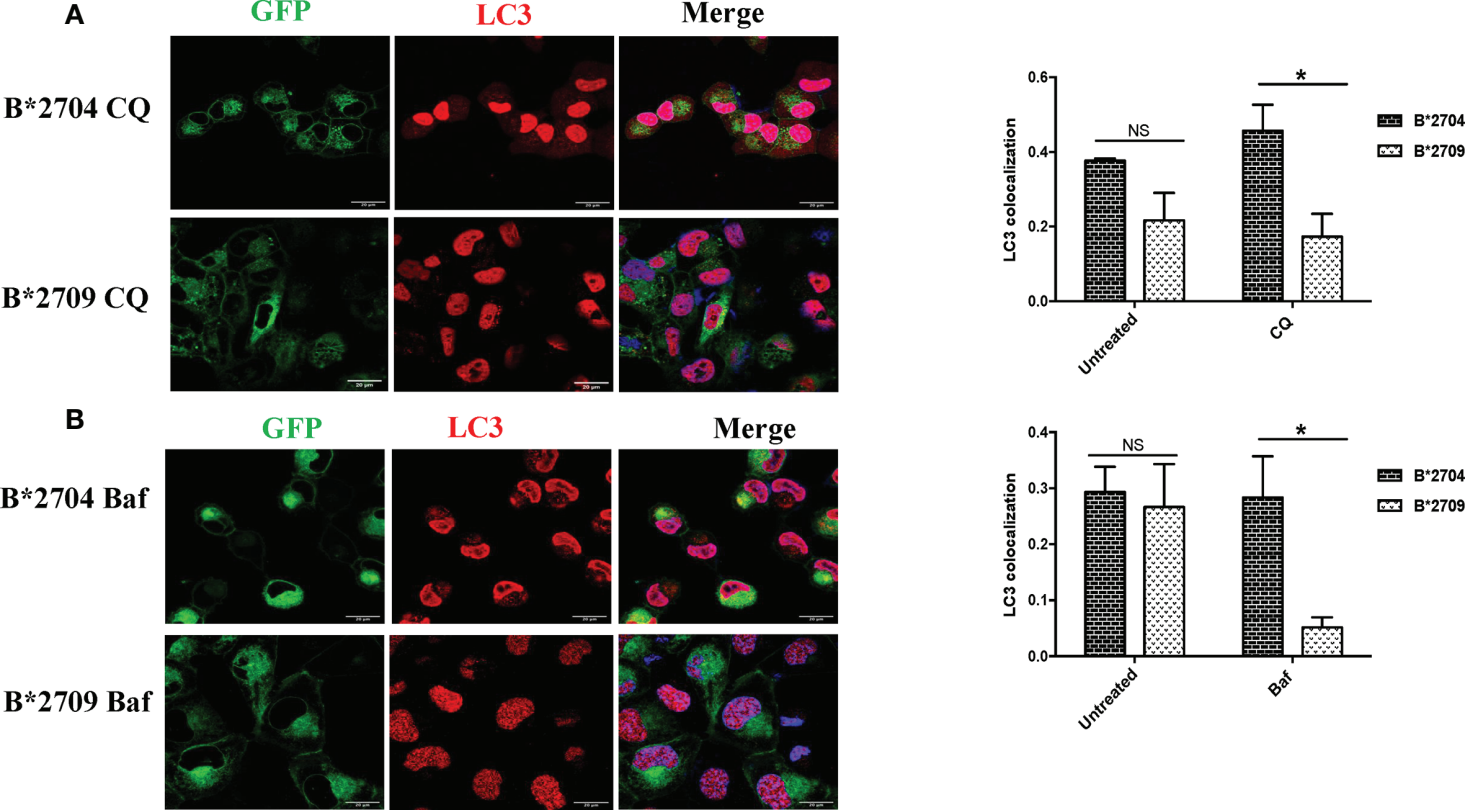
Cells transfected with GFP fusion constructs of the disease-associated, B*2704, and non-disease-associated, B*2709, subtypes were treated with chloroquine (CQ) **(A)** and bafilomycin (Baf) **(B)**. Immunofluorescence staining was performed by confocal microscopy using anti-LC3 antibody (probed with anti-rabbit Alexa Fluor 568) (red). Nuclear staining was done using DAPI (blue). Colocalization was determined using ImageJ software through Pearson’s coefficient value. Scale bar, 20 μm. GFP, green fluorescent protein. NS, non significant (p > 0.05); *p < 0.05.

**Figure 5:**
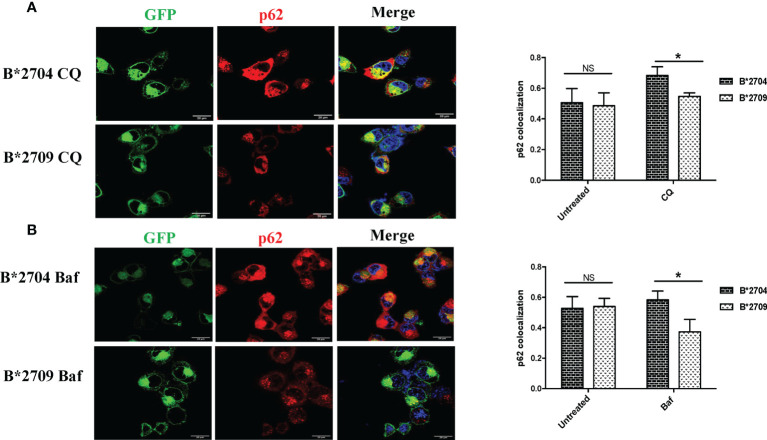
Cells transfected with GFP fusion constructs of the disease-associated, B*2704, and non-disease-associated, B*2709, subtypes were treated with chloroquine (CQ) **(A)** and bafilomycin (Baf) **(B)**. Immunofluorescence staining was performed under confocal microscopy using anti-p62 antibody (probed with anti-rabbit Alexa Fluor 568) (red). Nuclear staining was done using DAPI (blue). Scale bar, 20 μm. GFP, green fluorescent protein. NS, non significant (p > 0.05); *p < 0.05.

Interestingly, when the cells were treated with CQ, significantly increased binding of HC10 was observed (p = 0.04) in the case of B*2709-expressing cells (unlike that seen in cells transfected with B*2704), as shown in **Figure 3A**. The increased HC10 reactivity following CQ treatment in B*2709 is intriguing, given that the autophagy markers were significantly more in cells expressing B*2704 and not in B*2709-transfected cells (as also expected from the proteomics-based data) (**Figures 3C, D**). This was rationalized as follows: the drug CQ is generally used as an autophagy inhibitor, but recently, it has been found that it can also inhibit the endosomal–lysosomal pathway (16). Therefore, we hypothesize that in the case of cells expressing B*2709, the increased signal of HC10 upon CQ treatment could have arisen as a consequence of CQ-induced inhibition of the endosomal–lysosomal pathway (16). This was in line with the proteomics data (above) with confirmation of the involvement of this pathway in B*2709 cells, which if inhibited by CQ would be expected to lead to decreased clearance of aggregated species of B*2709 with a consequent increase in HC10 reactivity. Confocal microscopy was also carried out to analyze the autophagy markers LC3 and p62 (**Figures 4**, **5**, respectively; **Figure S6**) using both inhibitors CQ and Baf.

In B*2704-expressing cells, CQ (**Figure 4A**) and Baf (**Figure 4B**) treatments led to increased expression of LC3 puncta, with colocalization of GFP and LC3 signals. Statistically increased colocalization of signals of GFP and LC3 was observed in B*2704-transfected cells vs. B*2709-transfected cells upon treatment with CQ (p = 0.03) as well as with Baf (p = 0.02).

Since, both CQ and Baf increase the pH within the lysosomes, the autophagic degradation is compromised (15, 17), with the resulting accumulation of autophagic vacuoles. Such LC3-positive autophagic vacuoles are more evident in B*2704-expressing cells than in the case of B*2709-expressing cells, suggesting impairment of an ongoing autophagic process in the former (**Figure 4**).

We also evaluated the effects of these inhibitors on the expression of p62, which is degraded by autophagy (**Figure 5**; **Figure S6**). Treatment with CQ and Baf results in increased colocalization of GFP (originating from HLA-B27 heavy chains) and p62 in B*2704-expressing cells as compared with B*2709-transfected cells (p = 0.03 and p = 0.02, respectively).

### Aggresomal Pathway in B*2704

**Figure 6:**
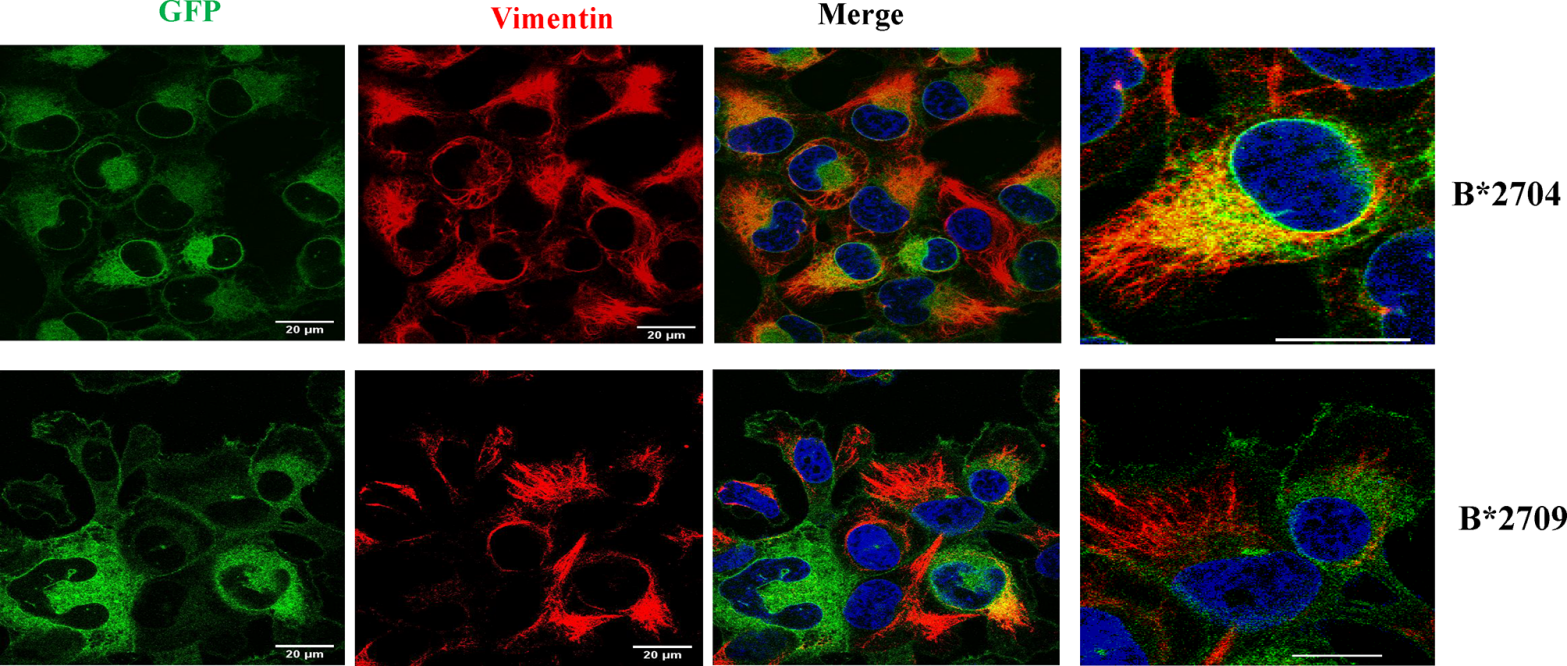
Representative confocal microscopy images of cells transfected with GFP-fusion (green) constructs of B*2704 and B*2709 subtypes, using anti-vimentin antibody (probed with anti-mouse Alexa Fluor 594; red). Nuclei were stained with DAPI (blue). Merged images of GFP with vimentin, as well as vimentin with DAPI, are also shown. Scale bar, 20 μm. Images shown on the right are enlarged views of a section of the merged image. GFP, green fluorescent protein.

To understand the role of the aggresomal pathway in B*2704 (as suggested by Gene Ontology software analyses), we checked the expression of the intermediate filament protein, vimentin, which is known to form a cage-like structure surrounding aggresomes (18, 19). An observation of the GFP signal together with DAPI staining (for nuclei) showed that HLA B*2704-transfected cells formed perinuclear aggresome-like structures, which were absent in B*2709-transfected cells (**Figure 6**). Colocalization studies showed that, unlike the B*2709-transfected cells, the GFP signal colocalized with vimentin in the perinuclear region in the B*2704-expressing cells, indicative of aggresome formation (**Figure 6**).

### Unfolded Protein Response Involvement in B*2704-Transfected Cells and Proteasomal Pathway Involvement in B*2709-Transfected Cells

**Figure 7:**
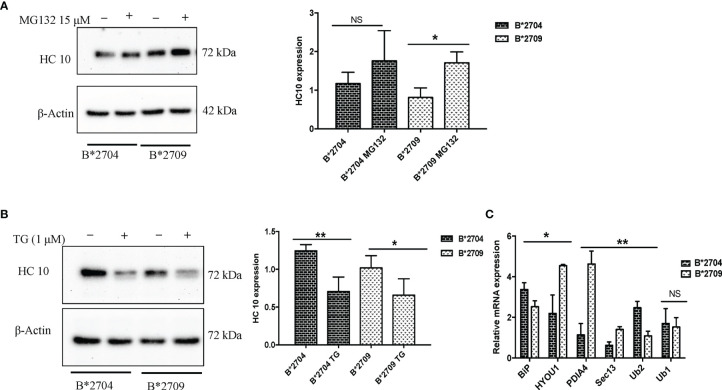
Stable transfectants of pEGFP-HLA-B27 subtypes (B*2704 and B*2709), untreated (− or UT), or treated with MG132 (+) **(A)** or thapsigargin **(B)**. The blots were probed with HC10. Actin levels were probed to serve as loading control for all samples and allow normalization of the signals. In each panel, histograms show densitometry data (mean ± SD) from 3 experiments corresponding to the Western blotting, using ImageJ software. Real-time PCR was carried out for expression of UPR markers, BiP, ubiquilin-1 and ubiquilin-2, and vesicle transport markers HYOU, PDI, and SEC13 **(C)**. Histogram shows data (mean ± SD) from 3 experiments for each marker. NS, non significant (p > 0.05); *p < 0.05; **p < 0.01.

To assess the role of proteasome-mediated degradation and UPR in degradation of HLA-B27 aggregates, we used, respectively, the proteasomal inhibitor (MG132) and an inducer of UPR, TG (inhibitor of the Ca^2+^ pump in ER), individually, i.e., in separate experiments on the respective transfected cells. The formation of aggregates was assessed by HC10 staining on Western blotting. Treatment with MG132 resulted in significantly increased binding of HC10 in B*2709-transfected cells (unlike that observed with B*2704-transfected cells), suggesting that the aggregates in the case of the B*2709-transfected cells are actively degraded by the proteasome (**Figure 7A**). Following the treatment with the UPR inducer, TG (inhibitor of the Ca^2+^ pump in ER), significantly decreased binding of HC10 was observed in samples of both the subtypes; however, the reduction was observed to be greater with the disease-associated subtype (B*2704) than with the non-disease-associated subtype (B*2709) (**Figure 7B**), implying a better UPR response by the former than the latter. One of the three UPR programs employed by cells to regulate cellular homeostasis is the attenuation of *de novo* protein synthesis through phosphorylation of the protein translation initiation factor 2 (eIF2α) (20). An examination of the proteomics data revealed that this was the case for B*2704, wherein eukaryotic translation initiation factor 2 subunits 1 and 2, and eukaryotic translation initiation factor 2A were increased, as compared with cells expressing B*2709 after transfection, by 1.68-fold, 1.59-fold, and 1.65-fold, respectively (**Table S2**).

In order to confirm and compare the UPR response in the two subtypes, the two types of transfected cells were treated with MG132, and phosphorylation of eiF2α was assessed by Western blotting. It was observed that the expression of phospho-eIF2α was more in the case of B*2704 as compared with B*2709 (**Figure S7**), confirming once again that UPR plays an important role in the former.

Additionally, real-time PCR was carried out for the two types of transfected cells, for validation of some of the important proteins identified through the proteomics study. The expression of BiP and ubiquilin-1 was significantly increased in B*2704, as compared with B*2709 (**Figure 7C**), implying the role of UPR and autophagy in the former subtype. A significant increase in expression was observed for SEC13, PDI, and Hyou1 in B*2709-transfected cells (**Figure 7C**), confirming the importance of vesicle transport or endosomal pathway in these cells. **Table 4** lists the comparative fold-changes of proteins, as determined by proteomics and real-time expression studies.

**Table 4:** Comparison of fold change of protein expression for selected proteins, determined by proteomics-based evaluation as well as by real-time PCR analysis.

Protein	Fold change in proteomics	Fold change in real-time PCR	HLA B-27 subtype
BiP	1.67	3.37	B*2704
Ubiquilin-1 (UBQLN1)	1.62	1.69	B*2704
Ubiqilin-2 (UBQLN2)	1.5	2.47	B*2704
Sec 13	2.73	1.4	B*2709
Hypoxia upregulated (Hyou1)	1.64	4.63	B*2709
Protein disulfide isomerase family A (PDIA4)	1.78	4.5	B*2709

## Discussion

Out of the >160 known subtypes of HLA-B27 (21), some are disease associated, such as B*2704 and B*2705, while others are non-disease associated, e.g., B*2709 and B*2706. Among these well-known subtypes, B*2704 is strictly associated with the disease, while B*2705 is sometimes not associated with the disease (22), and the disease-association status of B*2706 has been controversial, mainly because it is a rare subtype, and therefore, there are only a few studies reported for HLA-B*2706. This has made it difficult to ascribe the association status of B*2706 with certainty (23). These observations dictated our choice of the two reliable representatives of the disease-associated and non-disease-associated subtypes, and accordingly, we decided to focus on B*2704 as a definite representation of the disease-associated subtype and B*2709 as a non-associated subtype.

It has been previously well documented that fusion of fluorescent proteins to various HLA-B27 subtypes does not interfere with their interaction with β2m, or export to the plasma membrane in a fully folded conformation; such fusion proteins of different subtypes of HLA-B27 display comparable levels of ME1- and HC10-reactive HLA-B (3). Therefore, we performed a comparative and unbiased analysis of the proteomes of cells expressing GFP fusion constructs of the disease-associated HLA chains (B*2704) and non-disease-associated HLA chains (B*2709), which allowed us to decipher the operating QC mechanisms in both subtypes.

Misfolded proteins in the ER trigger a stress response, called the UPR, which tries to restore normal cellular homeostasis through the activation of one or more of the three sensors: IRE1α, PERK, and ATF6 (24). Cellular homeostasis by UPR occurs through i) decrease in *de novo* protein synthesis, or ii) by increasing the folding efficiency of proteins by inducing the expression of chaperones, or iii) by increased degradation of the misfolded proteins *via* the ERAD pathway through ubiquitination and processing in the proteasomal system. ERAD in turn can regulate other QC systems such as UPR and autophagy (and *vice versa*) (25). It is pertinent to note that UPR signalling does not necessarily invoke all three arms of the UPR pathway (26). For example, prolonged ER stress in neurodegenerative diseases leads to activation of UPR signalling, triggering a set of pro-death programs (27); however, it has been seen that XBP1 generation through splicing (involved in IRE1 signalling) is dispensable in the process and does not contribute to the neurodegeneration associated with prion protein misfolding (28). In our study too, we did not observe any splicing of XBP1 in B*2704 or B*2709-transfected cells. Further, there was no difference in the level of splicing observed between the two types of cells upon treatment with MG132, implying that in this case too, XBP-1 is dispensable in the UPR pathway (data not shown).

Label-free quantitation data comparing upregulated proteins in B*2704-transfected cells (with respect to B*2709-transfected cells) showed upregulation of the following six proteins related to UPR: i) BiP, ii) phospho-eIF2, iii) ubiquilin-1, iv) ubiquilin-2, v) sequestosome, and vi) Rab7. BiP or HSPA5 is a master regulator for ER stress, which controls the activation of UPR signalling. It is required for ER integrity and ER stress-induced autophagy (29). Phosphorylated eIF2 represses the translational machinery in response to stress through the UPR process. Thus, the overexpression of BiP and phospho-eIF2α in B*2704 indicates activation of UPR. It may be noted that the phosphorylation of eIF2α has also been shown to be a central event for the stimulation of autophagy (30). Accordingly, upregulation of ubiquilin-1 (UBQLN1) and ubiquilin-2 (UBQLN2) in B*2704-transfected cells was observed. The ubiquilins are thought to play a role in regulating the maturation of autophagy and expanding the involvement of ubiquitin-related proteins in autophagy; *UBQLN2* is implicated in macroautophagy through its indirect interaction with LC3 (14). These proteins are also important components of the protein QC process, regulating different protein degradation mechanisms and pathways, including the ubiquitin–proteasome system (UPS) and the ERAD pathway. UBQLN2 plays a role in ERAD, by its binding to ubiquinated substrates to drive degradation by the 26S proteasome (31). UBQLN1 has been reported to be associated with Alzheimer’s disease (32), while UBQLN 2 has gained importance because of its association with amyotrophic lateral sclerosis and frontotemporal dementia (ALS/FTD) pathogenesis (33). Sequestosome or ubiquitin-binding protein p62 is an autophagy adapter protein that binds to the target (cargo) and undergoes LC3-mediated delivery to the autophagosomes for lysosomal proteolysis (34). Finally, RAB7 is a member of the family of GTPases present on the late endosomes, which is crucial for complete autophagic flux (35, 36). Therefore, increased expression of all six proteins clearly points towards UPR and autophagy playing a predominant role in the clearance of aggregates in B*2704-transfected cells.

During the initiation of autophagy, LC3-I is cleaved and lipidated to form LC3-II; p62 is another autophagy marker, which is sequestered within autophagosomes, followed by degradation by lysosomes (37). Treatment with CQ induces the formation of autophagosomes, with inhibition of autophagosome degradation in the later stage of autophagy (38). The decrease in HC10-specific forms of B*2704 observed in the presence of CQ may be justified on the basis of the fact that although CQ treatment inhibits autophagy, it can induce ER stress as well as UPR through the PERK-eIF2α-ATF4 pathway (39), so that the clearance of aggregated forms of the protein may be UPR-driven. This was indeed found to be so, as exemplified by the increase in activation of UPR genes (by real-time PCR) and phosphorylation of eIF2α. Using a potent inhibitor like Baf led to significantly increased accumulation of heavy chains of B*2704-expressing cells, clearly indicative of autophagic pathway. The difference in levels of accumulation of HC10 reactive species upon treatment with CQ and Baf may result from the fact that although both CQ and Baf cause changes in intra-lysosomal pH change, the mechanisms of inhibition for the two inhibitors are not the same (16).

Interestingly, however, in the case of B*2709-transfected cells, treatment with CQ led to increased aggregate formation (HC10 reactivity). Since CQ has been shown to induce an autophagy-independent severe disorganization of Golgi and endo-lysosomal networks (16), we propose that the increase in the HC10 signal, following treatment with CQ, occurs because of impairment of vesicle fusion, or due to disruption of the endosomal lysosomal pathway (11) in which proteins are received from ER in the form of vesicles, and internalized with help of receptors present on the membranes of vesicles (which fuse with late endosome and finally with lysosomes where aggregates are degraded by the acidic pH).

A comparison of LC3 levels in transfected cells in the absence and presence of CQ showed that LC3 levels increased in both B*2704 and B*2709-transfected cells upon CQ treatment. This was expected in the former (because of the autophagy pathway operating in these cells as discussed above) but not in the latter and was somewhat puzzling. This is rationalized on the basis of the new concept that has emerged over the last few years, referred to as “non-canonical autophagy” involving CQ-induced parallel induction of single-membrane endolysosomal LC3 lipidation, which is proposed to regulate the fusion of vesicles with lysosomes (40, 41). We believe that in the case of B*2709-transfected cells, where removal of aggregates of misfolded HLA chains occurs through the endosomal–lysosomal pathway, such an increase in LC3 would be entirely expected.

The observation of aggresome formation in B*2704-expressing cells was expected since clearance of misfolded proteins accumulated in the aggresomes also occurs through the macroautophagy pathway (42), already determined to be operating in these cells. Another result to support the formation of aggresomes was the proteomics study-based observation of increased expression of proteins associated with the dynein–dynactin system (upregulation by 1.54-fold of dynein light chain roadblock-type 1 or DYNLRB1) in B*2704-transfected cells, which plays a critical role in the retrograde transport of cargo of misfolded proteins, along microtubules, for processing at perinuclear aggresomes (43). Also, SQSTM1 (or p62) and the Hsp70 co-chaperone, BAG3, are proteins known to be associated with dynein–dynactin (12, 13); p62 scaffolding protein is required for dynein function in trafficking cargo to the perinuclear region of the cell along the degradative pathways (13), and BAG3 is involved in chaperone-based aggresome targeting, which is independent of substrate ubiquitination (12). p62 and BAG3 were enriched in B*2704-transfected cells by 1.5-fold and 1.73-fold, respectively (as listed in **Table 3**). Some of the other proteins involved in retrograde transport were also upregulated in B*2704; these include synaptosomal-associated protein 29 (44) ubiquilin-1 (45), early endosome antigen 1 (46), and the dynamin-binding protein (43, 47) (**Table 3**), supporting the retrograde transport of cargo to aggresomes.

From all of the above, it is clear that in B*2704-transfected cells, besides UPR, macroautophagy (involving the generation of a double-membrane autophagosome, which forms non-selectively around bulk cytoplasm) may cause carriage of the misfolded B*2704 protein molecules through a series of vesicular fusion events into the lysosomes for subsequent pH-dependent degradation by lysosomal hydrolases (48).

There could be several reasons for the failure of UPR and autophagy to clear the B*2704 aggregates. Degradation pathways adopted by aggregates can vary, depending on whether the protein is in soluble or fibrillar form and whether it bears any post-translational modifications. It is possible that the aggregates formed by B*2704 are more stable and resistant to degradation; indeed, our previous studies have hinted at this possibility (6) since the α1 and α2 domains of B*2709 are less stable to chemical and thermal denaturation than the corresponding domains of B*2705 (49). Such long-lived proteins would be expected to follow the autophagic pathway rather than the proteasomal machinery, which is more suited for short-lived proteins (50). In any case, such aggregates would not be able to pass through the proteasome. It is also possible that such aggregated proteins would resist being dissociated easily from the components of the autophagic pathway before being delivered to the lysosome. Although autophagy appears to operate to clear the B*2704 aggregates, the kinetics of the clearance mechanism may not be suited to the number of aggregates and the rate of autophagic clearance. It is also possible that the aggregates are resistant to the low pH of lysosomes, resulting in a buildup of protein in lysosomes (akin to the lysosomal storage disorders) (51). This possibility will need further examination.

Accumulation of misfolded forms of B*2704, including dimers and oligomers or HMW aggregates, is expected to cause increased ER stress and UPR. In the case of dimers, several studies have proposed that interaction of such dimers with natural killer (NK) cells, myelomonocytes, and lymphocytes, can result in stimulation and proliferation of KIR3DL2+ CD4+ T lymphocytes, with consequent production of proinflammatory cytokines such as IL-23 and IL-17; these cytokines can further collaborate with TNF-α or IFN-γ, thereby causing sustained immune activation with effects on bone construction and contributing to the disease pathogenesis (4, 5, 52, 53). We propose that HMW species of B*2704 may behave in a similar manner.

In the case of B*2709-transfected cells, proteomic data analyses using available software packages suggested that the proteomes of B*2709-transfected cells are enriched in proteins facilitating vesicle-mediated transport from the ER to Golgi, COPII vesicle coating, vesicle transport, receptor-mediated endocytosis, and the proteasomal pathway (**Tables S4, S5**). These processes are facilitated by proteins such as SEC13, SEC31, heat shock protein 70 (Hsp70), and PDIA4 found to be upregulated in B*2709 cells (**Table S3**). The SEC13/SEC31 proteins are required for the generation of COPII vesicles, which direct the budding of vesicles from the ER and play an important role in the anterograde transport of proteins to Golgi (54). Hypoxia upregulated protein 1 (or Hyou1) belongs to the Hsp 70 family and was found to be upregulated by 1.64-fold in B*2709; it plays an important role in protein folding and secretion in the ER (55). PDIA4 belongs to the protein disulfide isomerase (PDI) family of ER proteins that catalyze protein folding and thiol–disulfide interchange reactions (56).

Previous studies have demonstrated evidence of HLA-B27 misfolding in the gut of AS patients and activation of autophagy (but not UPR) regulating intestinal modulation of IL-23 (57). Autophagy in combination with ERAD has also been suggested to play a role in the clearance of excess HLA class I heavy chains expressed in transgenic rats (58). Further, there are other studies implicating the importance of UPR as an intracellular stress response pathway (induced by misfolded forms of HLA-B27 in B27/hβ2m transgenic rats) (59, 60), responsible for causing an innate immune activation (61), cytokine dysregulation, and increased production of proinflammatory cytokine axis (62, 63).

The novelty of this study is that this is the first proteomics-based study seeking to explain the differential disease association of the HLA-B27 subtypes with respect to differences in the mechanism of disposal of HMW aggregates of HLA-B27 molecules. The ER QC processes (such as ERAD and UPR), as well as autophagy, have been previously shown to bring about the elimination of HLA-B27 misfolded proteins, and our data also show that these pathways are important, but only as far as B*2704 is concerned. Here too, we additionally provide data to show the importance of aggresomes in garbage disposal, which are degraded by autophagy upon transport to an autophagosome. On the other hand, it is noteworthy that the disposal of misfolded species in the non-disease-associated B*2709 subtype occurs mainly through the endosomal–lysosomal pathway, which has not been talked about as yet in the field of spondyloarthropathies.

This observation of a stark difference in disposal mechanisms involving B*2704 and B*2709 not only will aid in better understanding of AS disease pathogenesis but could also provide insights into the better design of therapeutics, e.g., along the lines of diseases such as multiple myeloma, where a combinatorial approach of using proteasome, autophagy, and aggresome inhibitors was used to induce a cellular stress response of apoptosis (64, 65).

## Data Availability Statement

The original contributions presented in the study are publicly available and have been deposited to the ProteomeXchange Consortium via the PRIDE partner repository with the dataset identifier PXD027999.

## Author Contributions

Study design: ML-G. Acquisition of data: AT. Analysis and interpretation: AT and ML-G. Manuscript preparation: AT and ML-G. Statistical analysis: AT and ML-G.

## Funding

This work was supported by a grant from the Department of Biotechnology, Government of India (BT/PR28516/MED/30/2022/2018).

## Conflict of Interest

The authors declare that the research was conducted in the absence of any commercial or financial relationships that could be construed as a potential conflict of interest.

## Publisher’s Note

All claims expressed in this article are solely those of the authors and do not necessarily represent those of their affiliated organizations, or those of the publisher, the editors and the reviewers. Any product that may be evaluated in this article, or claim that may be made by its manufacturer, is not guaranteed or endorsed by the publisher.

## Supplementary Material

Supplementary Figure 1 Boxplots representing abundance distribution of proteins in cells expressing B*2704 (blue colour) and B*2709 (orange colour) **(A)**, made using Proteome Discoverer (version 2.4). Principal Component Analysis (PCA) carried out using R software (version 3.4.1) **(B)**.

Supplementary Figure 2 Protein–protein interaction analysis (conducted by the String database, version 11.0) of 261 proteins found to be upregulated in cells expressing B*2704 **(A)**. The proteins related to Unfolded Protein Response **(B)** and autophagy pathway **(C)** are also shown.

Supplementary Figure 3 Protein–protein interaction analysis (conducted by the String database, version11.0) of 174 proteins found to be upregulated in cells expressing B*2709 **(A)**. The proteins related to vesicle transport from ER to Golgi are also shown **(B)**.

Supplementary Figure 4 Western blot corresponding to non-reducing SDS-PAGE gel electrophoresis of lysates derived from cells transfected with B*2704 and B*2709 under conditions of absence and presence of CQ. Blots were probed with HC 10 antibody to analyze the oligomeric species. Images were taken under short and long exposures to better highlight the presence of differently oligomerized species in each case.

Supplementary Figure 5 Real Time PCR was carried out to evaluate the expression of UPR markers for non-transfected cells (H1299) and transfected cells expressing B*2704 and B*2709, following treatment with CQ (50μM) for 12h.

Supplementary Figure 6 Immunofluorescence staining was performed for H1299 cells **(A)**, cells transfected with B*2704 **(B)** and B*2709 (**(C)** subtypes by confocal microscopy using anti-LC3 and anti-p62 antibody (probed with anti-rabbit Alexa Fluor 568) (red). Nuclear staining was done using DAPI (Blue).

Supplementary Figure 7 Stable transfectants of pEGFP-HLA-B27 subtypes (B*2704 and B*2709), treated with (+) or without (-) the proteasomal inhibitor, MG132, and probed with anti-phospho-eIF2α antibody.
