# Supplementary figures and images for: Differences in Cellular Clearing Mechanisms of Aggregates of Two Subtypes of HLA-B27
Source: Front Immunol. 2022 Jan 10;12:795053. doi: 10.3389/fimmu.2021.795053 (PMC8785436; doi:10.3389/fimmu.2021.795053)

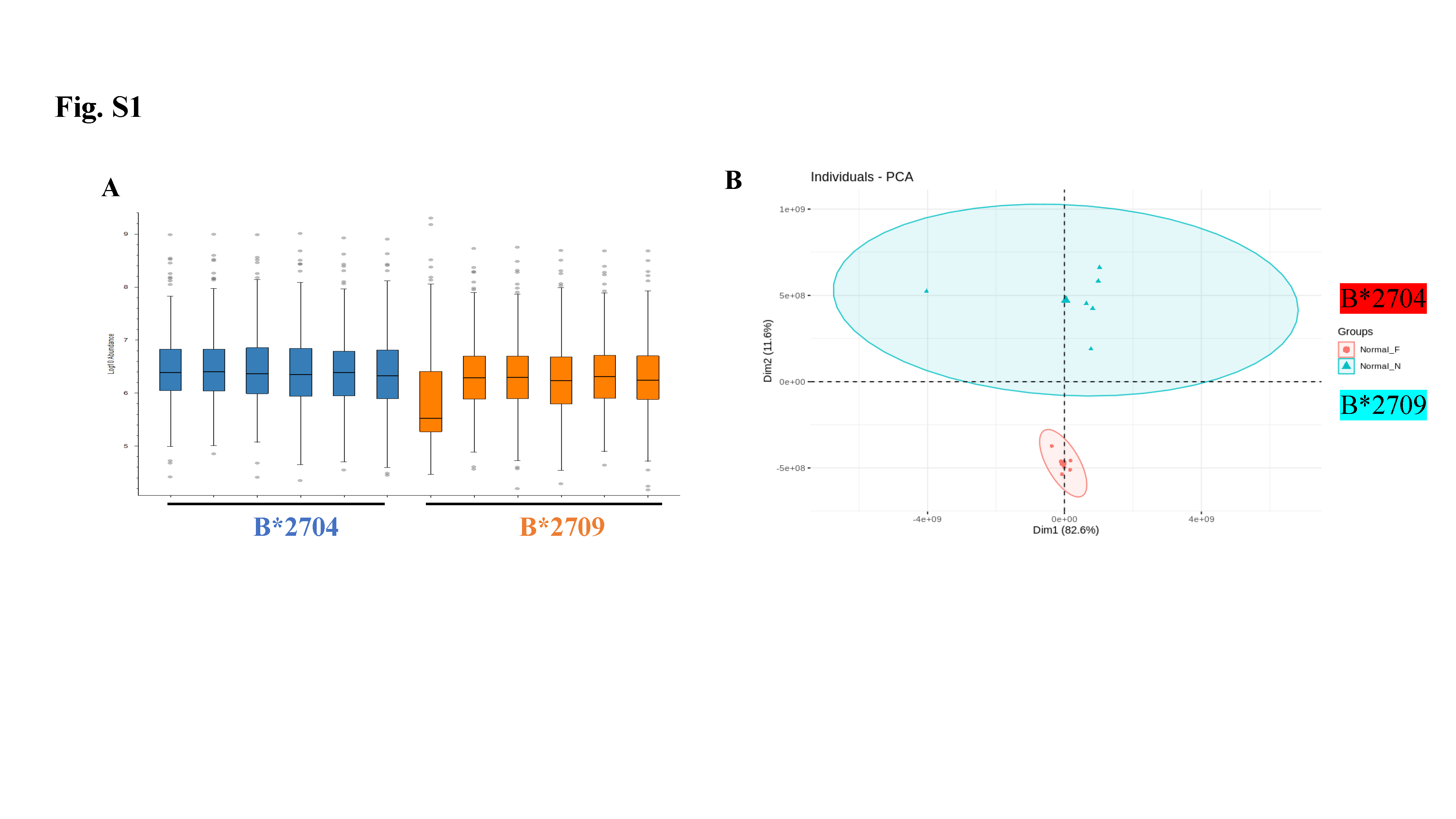

Supplement: Supplementary Figure 1 — Boxplots representing abundance distribution of proteins in cells expressing B*2704 (blue colour) and B*2709 (orange colour) (A), made using Proteome Discoverer (version 2.4). Principal Component Analysis (PCA) carried out using R software (version 3.4.1) (B). [file Image_1.tiff]

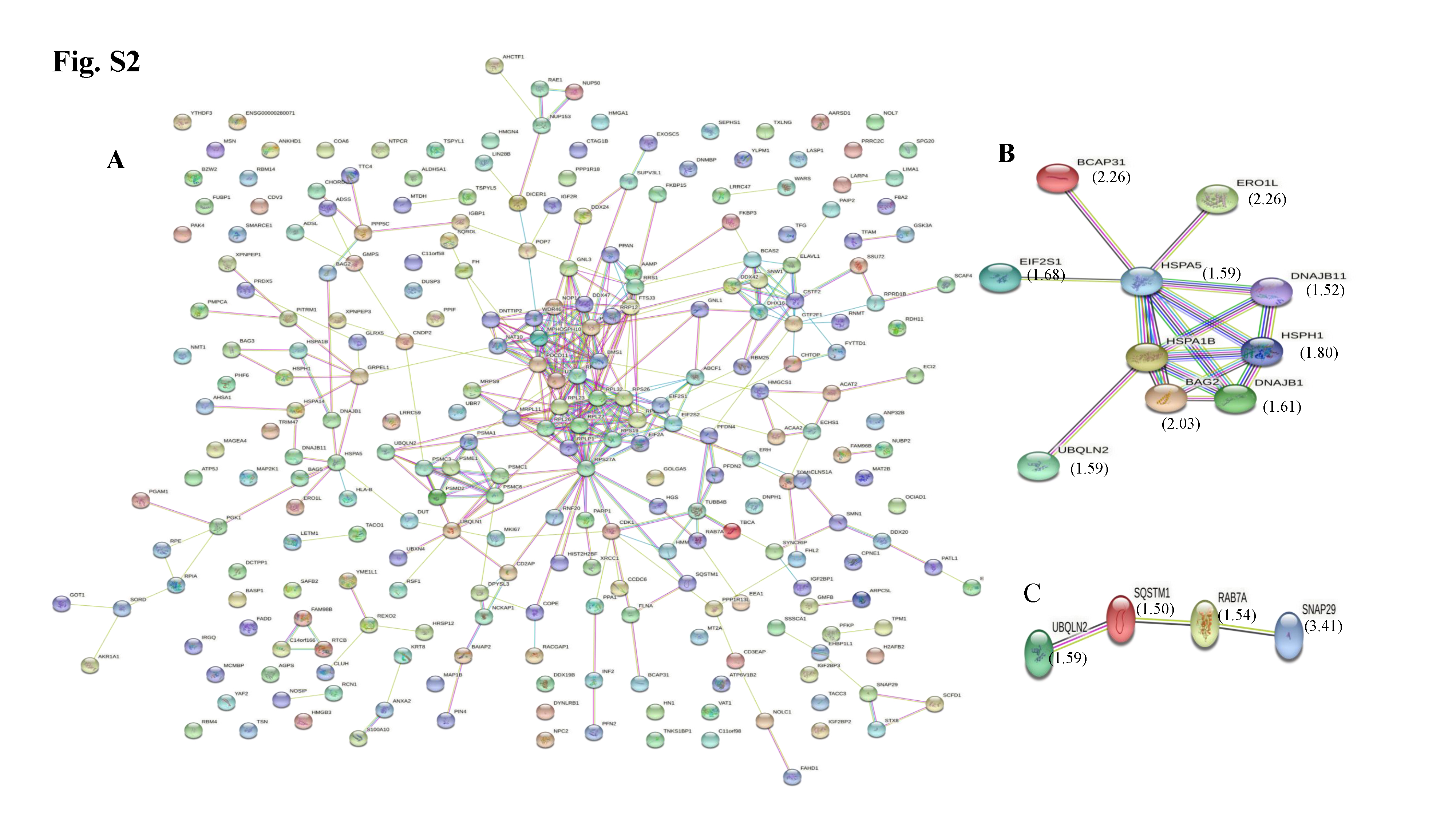

Supplement: Supplementary Figure 2 — Protein–protein interaction analysis (conducted by the String database, version 11.0) of 261 proteins found to be upregulated in cells expressing B*2704 (A). The proteins related to Unfolded Protein Response (B) and autophagy pathway (C) are also shown. [file Image_2.tiff]

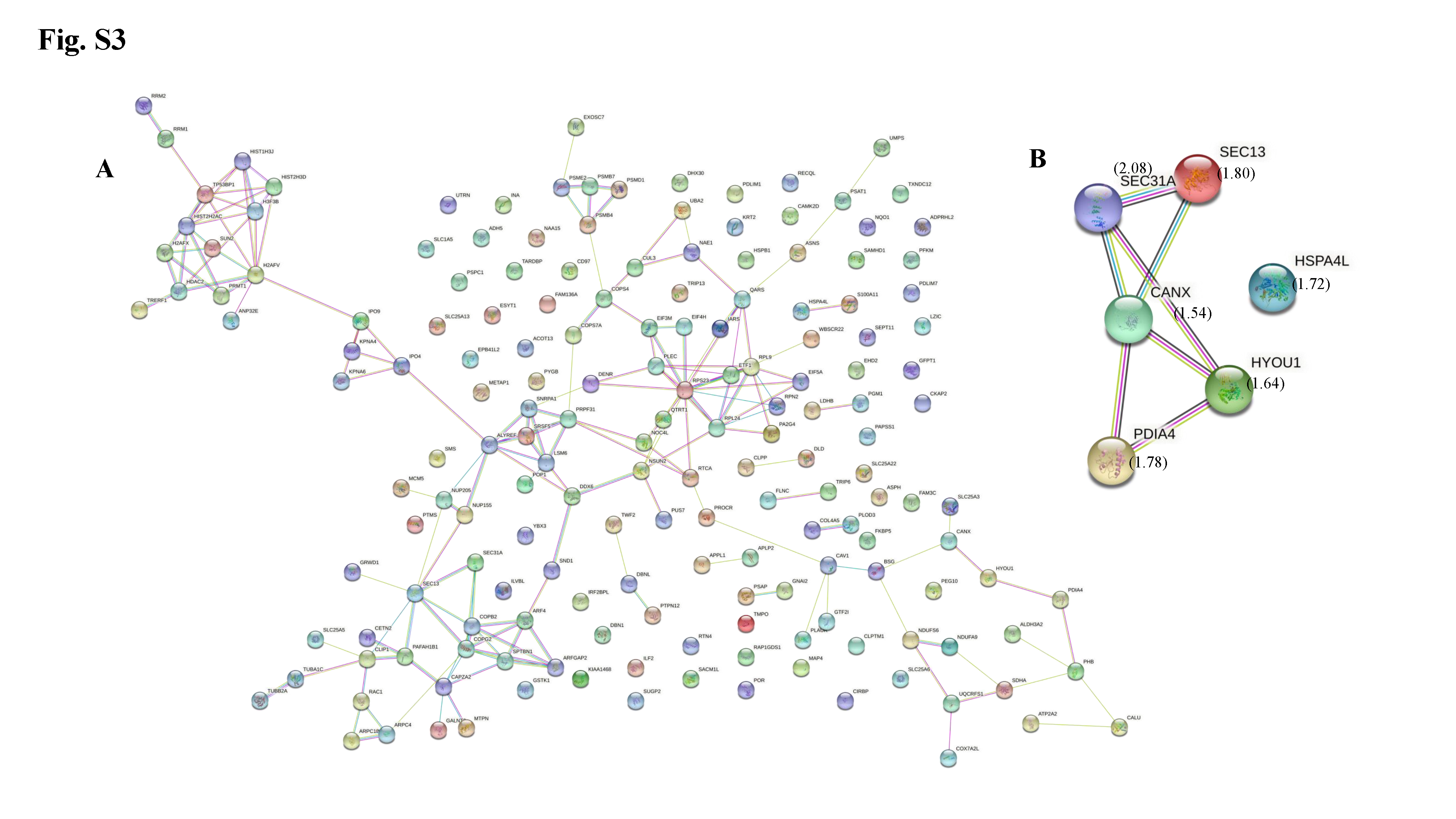

Supplement: Supplementary Figure 3 — Protein–protein interaction analysis (conducted by the String database, version11.0) of 174 proteins found to be upregulated in cells expressing B*2709 (A). The proteins related to vesicle transport from ER to Golgi are also shown (B). [file Image_3.tiff]

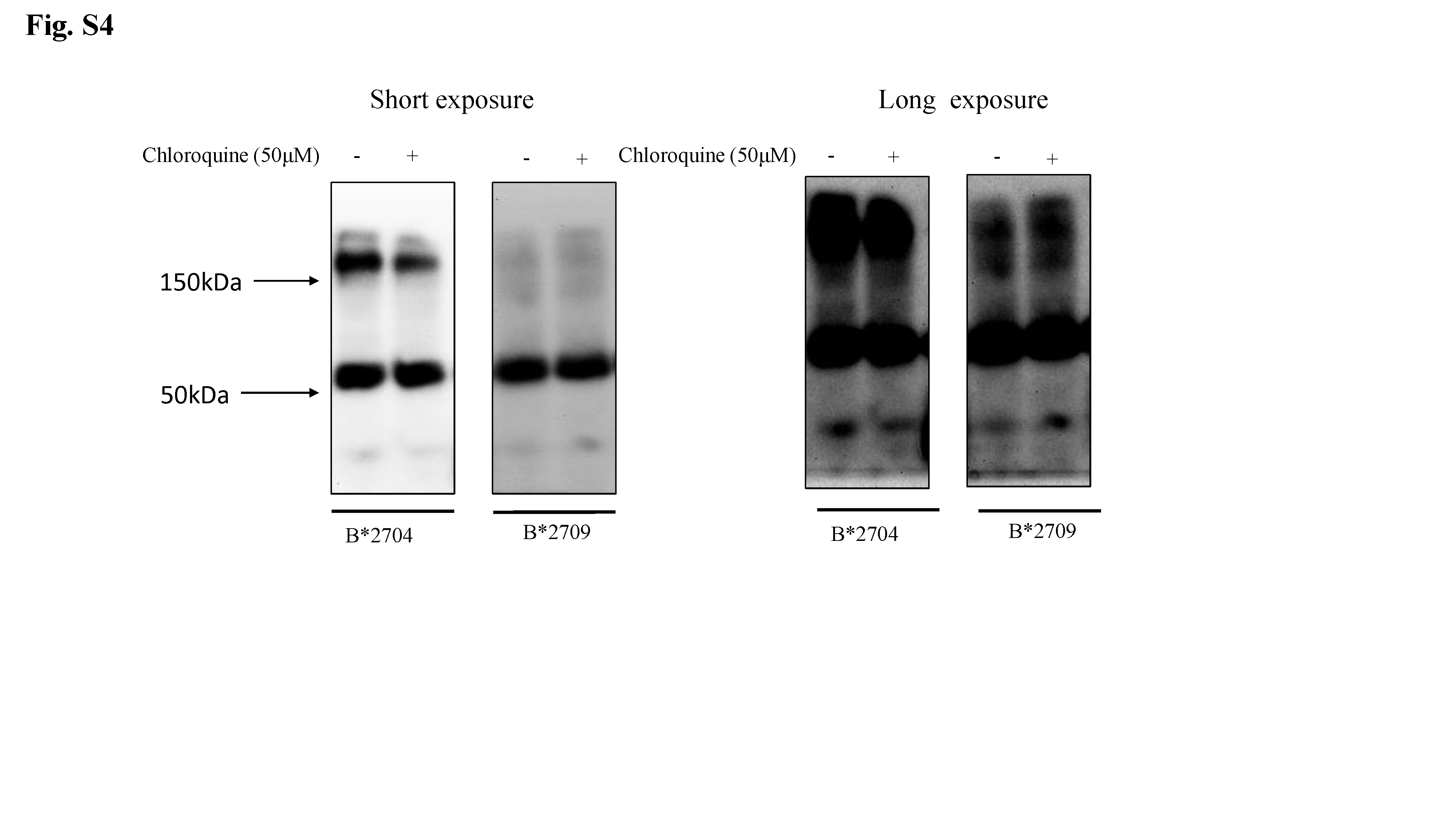

Supplement: Supplementary Figure 4 — Western blot corresponding to non-reducing SDS-PAGE gel electrophoresis of lysates derived from cells transfected with B*2704 and B*2709 under conditions of absence and presence of CQ. Blots were probed with HC 10 antibody to analyze the oligomeric species. Images were taken under short and long exposures to better highlight the presence of differently oligomerized species in each case. [file Image_4.tif]

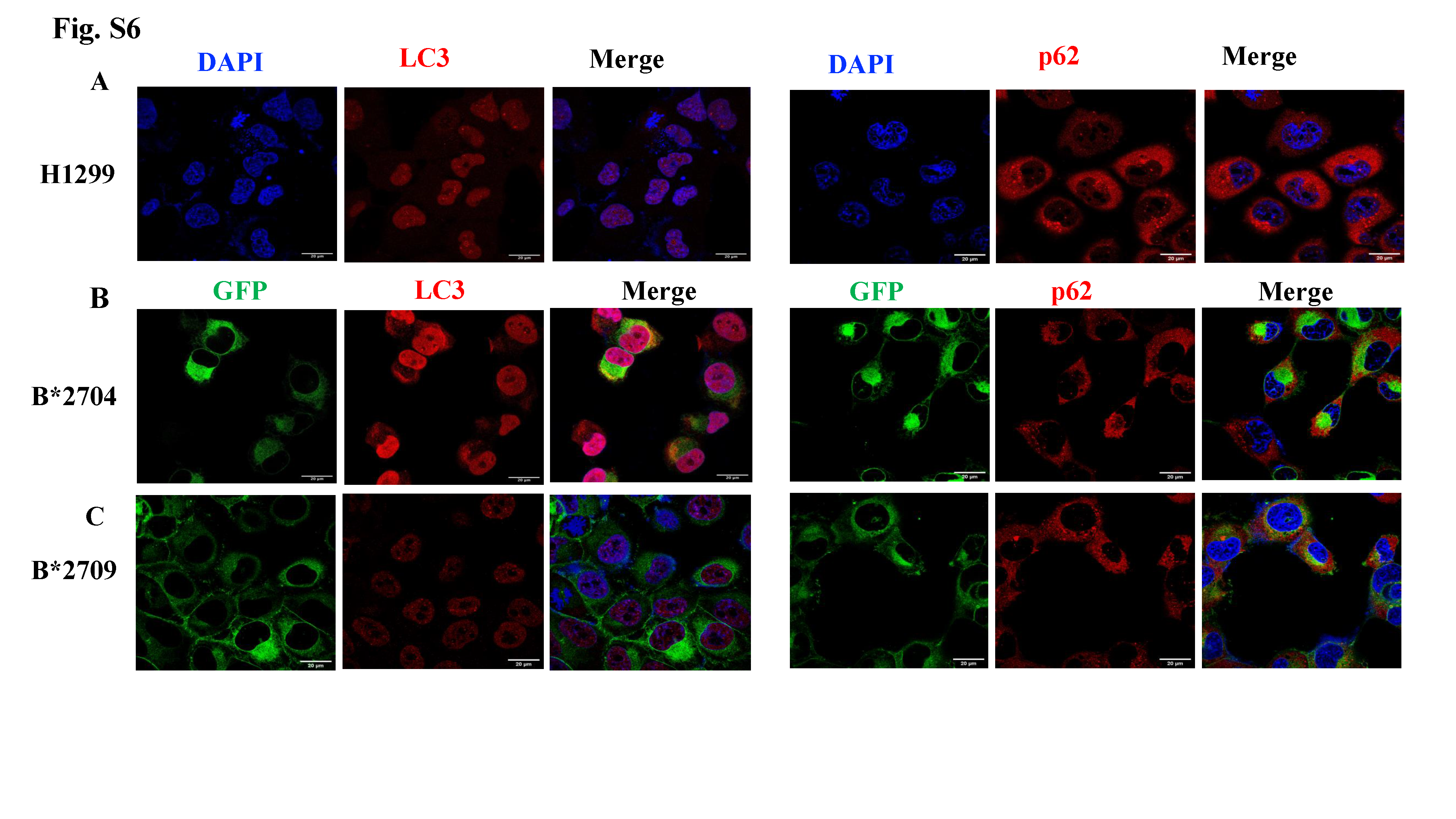

Supplement: Supplementary Figure 6 — Immunofluorescence staining was performed for H1299 cells (A), cells transfected with B*2704 (B) and B*2709 ((C) subtypes by confocal microscopy using anti-LC3 and anti-p62 antibody (probed with anti-rabbit Alexa Fluor 568) (red). Nuclear staining was done using DAPI (Blue). [file Image_6.tif]

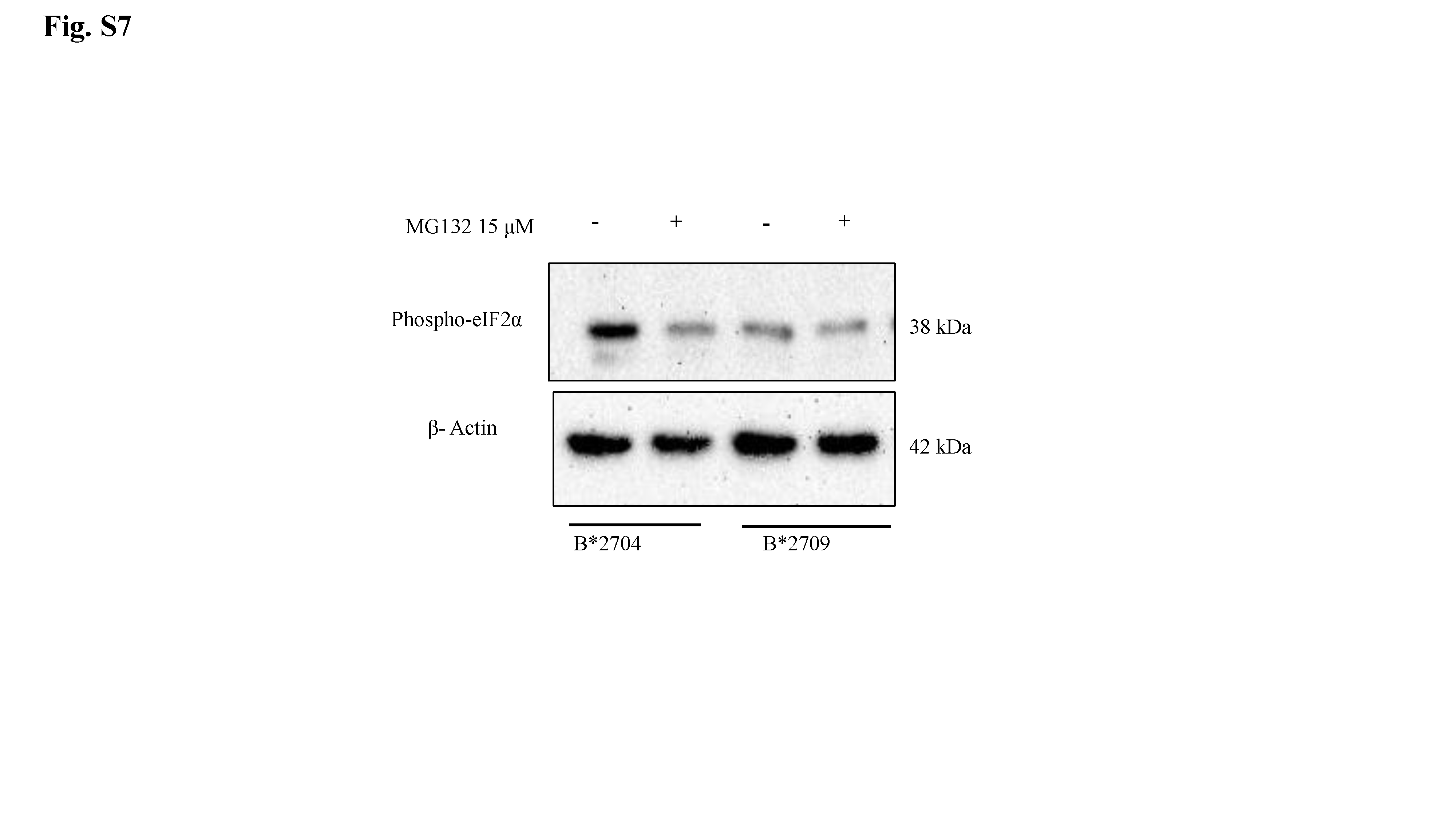

Supplement: Supplementary Figure 7 — Stable transfectants of pEGFP-HLA-B27 subtypes (B*2704 and B*2709), treated with (+) or without (-) the proteasomal inhibitor, MG132, and probed with anti-phospho-eIF2α antibody. [file Image_7.tif]
